# Transcriptional Evaluation of Neuropeptides, Hormones, and Tissue Repair Modulators in the Skin of Gilthead Sea Bream (*Sparus aurata* L.) Subjected to Mechanical Damage

**DOI:** 10.3390/ani14121815

**Published:** 2024-06-18

**Authors:** Rocío Piñera-Moreno, Felipe E. Reyes-López, Merari Goldstein, María Jesús Santillán-Araneda, Bárbara Robles-Planells, Camila Arancibia-Carvallo, Eva Vallejos-Vidal, Alberto Cuesta, María Ángeles Esteban, Lluis Tort

**Affiliations:** 1 Department of Cell Biology, Physiology and Immunology, Universitat Autònoma de Barcelona, 08193 Bellaterra, Spain; rocio.pinera@uab.cat; 2 Fish Health and Integrative Physiogenomics Research Team, Centro de Biotecnología Acuícola, Facultad de Química y Biología, Universidad de Santiago de Chile, Santiago 9170002, Chile; felipe.reyes.l@usach.cl (F.E.R.-L.); barbara.robles@usach.cl (B.R.-P.); camila.arancibia.ca@usach.cl (C.A.-C.); evallejos@udla.cl (E.V.-V.); 3 Centro de Nanociencia y Nanotecnología CEDENNA, Universidad de Santiago de Chile, Santiago 9170002, Chile; 4 Núcleo de Investigación Aplicada en Ciencias Veterinarias y Agronómicas, Facultad de Medicina Veterinaria y Agronomía, Universidad de Las Américas, La Florida 8250122, Chile; 5 Department of Cell Biology and Histology, Faculty of Biology, Campus Regional de Excelencia Internacional “Campus Mare Nostrum”, University of Murcia, 30100 Murcia, Spain; alcuesta@um.es

**Keywords:** *Sparus aurata*, skin wound, tissue repair, stress, neuro-immuno-endocrine axis, immune system, aquaculture

## Abstract

**Simple Summary:**

The fish’s skin is the tissue whose total area is in permanent contact with the aquatic environment. Despite its relevance, there are currently very few studies aimed at evaluating tissue repair mechanisms. In this study, we assessed the tissue repair response to mechanical damage in the skin of gilthead sea bream (*Sparus aurata*), revealing differences in the modulation of neuroimmune–endocrine response gene markers depending on the location of the lesion. Furthermore, wound healing in the ventral region was observed to be higher in magnitude and faster than in the dorsal region. We registered an increase in the expressions of hormone-related genes compared to the dorsal lesion. This research improves our understanding of the neuroendocrine and tissue repair response to mechanical damage in *S. aurata*. This study provides biologically relevant molecular markers for future studies of tissue repair.

**Abstract:**

The skin of bony fish is the first physical barrier and is responsible for maintaining the integrity of the fish. Lesions make the skin vulnerable to potential infection by pathogens present in the aquatic environment. In this way, wound repair has barely been studied in gilthead sea bream. Thus, this study investigated the modulation of peripheral neuro-endocrine and tissue repair markers at the transcriptional level in the skin of teleost fish subjected to mechanical damage above or below the lateral line (dorsal and ventral lesions, respectively). Samples were evaluated using RT-qPCR at 2-, 4-, and 20-days post-injury. Fish with a ventral lesion presented a trend of progressive increase in the expressions of *corticotropin-releasing hormone* (*crh*), *pro-opiomelanocortin-A* (*pomca*), *proenkephalin-B* (*penkb*), *cholecystokinin* (*cck*), *oxytocin* (*oxt*), *angiotensinogen* (*agt*), and (less pronounced) *somatostatin-1B* (*sst1b*). By contrast, fish with a dorsal lesion registered no significant increase or biological trend for the genes evaluated at the different sampling times. Collectively, the results show a rapid and more robust response of neuro-endocrine and tissue repair markers in the injuries below than above the lateral line, which could be attributable to their proximity to vital organs.

## 1. Introduction

The skin of a teleost is a non-keratinized living tissue that covers the animal’s entire body, protecting it from the entry of pathogens and the leakage of water, solutes, and nutrients. Like other vertebrates, it has a conserved organization consisting of the epidermis, dermis, and hypodermis. Fish skin, unlike mammals, has a layer of mucus that is secreted by the skin cells, acting as an additional external shield that provides them with a vast repertoire of defense characteristics, thus reflecting the adaptation of fish to the rich aquatic environment [1,2]. Due to this intimate environmental interaction, the skin in fish represents a protective mechanism, including elements of the crossroad between an innate immune response and the emergence of an adaptive immune response [3]. This immune response is carried out systemically and through mucosal surfaces, including the skin, intestine, gills, and nasopharynx, among others.

Fish are often subjected to different biotic and abiotic stressors in the environment. Current studies on gilthead sea bream (*Sparus aurata*) and trout (*Oncorhynchus mykiss*) suggest that the skin of fish also responds to stressors. This mechanism is thanks to the activation of the hypothalamic–pituitary–inter-renal (HPI) axis and the subsequent release of cortisol as the key mediator for the activation of the stress response. It also mediates the production of melatonin as an antioxidant. In daily aquaculture practice, it is very common for fish to be subjected to various environmental or imposed stressors (such as changes in the temperature, photoperiod, pH, oxygen saturation, population density, pathogen load, and virulence), which alter the temporal course of mucosal immune responses in a species-specific manner [4].

In response to stress, teleosts were the first vertebrates to develop a response that includes a complex network of signals involving the major regulatory systems: neural, endocrine, and immune [5,6], primarily through the HPI and the Brain–Sympathetic–Chromaffin (BSC) axes and their interaction with immune networks. The immediate secretion of corticosteroid-releasing hormone (CRH) stimulates the release of pro-opiomelanocortin (POMC), which is a pre-prohormone, a precursor to a diverse group of neuropeptides and pharmacologically active hormones, including adrenocorticotropic hormone (ACTH), melanocyte-stimulating hormone (α-MSH), and opioid peptides. Located in the anterior kidney are the inter-renal glands that synthesize cortisol and the chromaffin cells that synthesize catecholamines (adrenaline and norepinephrine) in response to a stressor. The activation of these mechanisms activates the stress response through the secretion of specific neuropeptides or hormones for each case [7]. Additionally, several studies have demonstrated that pro-inflammatory cytokines, such as interleukin (IL)-1, tumor necrosis factor (TNF-α), and IL-6, play an active role in regulating certain stress hormones (CRH and cortisol), demonstrating the interconnectedness of these regulatory systems and their role in fish physiology [6,8].

The skin is a dynamic tissue in which various systems (including the central nervous and endocrine systems) converge in response to a given stimulus [9,10]. An example of this multisystemic interaction is the response of the skin to a stressor. Thus, Slominski et al. [11] showed that the skin’s defense against stress is organized through a peripheral HPA action similar to the central HPA axis that regulates, coordinates, and executes local stress responses independently of the central axis. In addition, subsequent studies discovered that skin cells locally produce neurotransmitters such as POMC and CRH. They also express functional CRH receptors type-1 (CRH-R1) and ACTH in epidermal, dermal, and adnexal cells or release them in situ from skin nerve terminals. Likewise, annexed structures (apocrine and sebaceous glands and hair follicles) secrete exocrine skin products to strengthen the epidermal barrier, regulate thermoregulation, participate in defense against microorganisms, or social communication. Therefore, the mammalian skin constitutes a local neuroendocrine system, using similar mediators (neuropeptides and hormones) to those involved in the brain and pituitary endocrine systems, and its cells express functional receptors activated by neurotransmitters [11,12,13,14]. These neuroendocrine substances in the skin are derived from nerve fibers, and skin cells produce and secrete humoral signaling molecules (neurotransmitters, neuropeptides, and hormones). In addition, some neuropeptides can act as endogenous hormones or opioids, suppressing the sensation of pain and acting on the immune system [15]. A similar organization of the peripheral stress response is present in fish but modulated by the above-mentioned special characteristics of the mucosal surfaces of these aquatic animals [16].

In farmed fish, stress caused by skin injuries, wounds, abrasions, and ulcers can occur due to the higher densities and social interactions among individuals, causing hemorrhages and contamination or affecting the underlying tissues due to pathogenic organisms. Epidermal integrity is vital for fish defense. In fact, tissue alterations in the normal skin barrier function can allow for the colonization of commensal and opportunistic pathogenic microorganisms, which are always present in an aquatic environment [17]. The regenerative response following a skin injury in fish differs from that of mammals, where no blood clots are formed during the wound-healing process in fish. However, other phases are present: inflammation, re-epithelialization, new tissue formation, and remodeling [18]. All these changes in mucosal tissues produce local alterations of non-specific tissue receptors (glucocorticoid receptors increase in the leukocytes of the head kidney) and messenger substances such as neuropeptides, hormones (catecholamines such as CRH, and hypothalamic–pituitary–inter-renal axis stress corticosteroids such as cortisol), and cytokines (suppression of TNF-α, interferon (IFN)-γ, IL-2, and IL-12 and the production of IL-4, IL-13, IL-10, and transforming growth factor (TGF)-β), which activate the general physiological response [6,19]. Such processes involve a significant supply of energy and metabolic resources to support tissue repair formation.

In fish, the evaluation of skin repair has been scarcely evaluated. Some studies have assessed the effects of diets on the healing process of skin wounds [20] and skin re-epithelization, including the evaluation of gene markers associated with the extracellular matrix (ECM) and humoral and cellular elements of the immune response [21]. On the other hand, few reports are devoted to evaluating the potential differences in wound repair in different regions limited by the lateral line of the skin. At the gene expression level, there is evidence in sea bream of a greater modulation in the ventral than the dorsal region of a set of immune-related genes [1,22], suggesting a differential modulatory mechanism in response to wound healing depending on the skin region affected. However, there are no antecedents regarding the expression profile of peripheral neuro-endocrine and tissue repair markers in different skin regions subjected to mechanical damage. For this reason, in this study, we investigated the modulation of peripheral neuro-endocrine and tissue repair markers at the transcriptional level in the skin of teleost fish subjected to mechanical damage in gilthead sea bream. For this purpose, a set of genes encoding the molecules associated with these processes was evaluated. We analyzed the expression profiles in fish subjected to a lesion above (IU group) or below (ID group) the lateral line to identify molecular agents that are biologically relevant in the repair of the injury.

## 2. Materials and Methods

### 2.1. Fish and Rearing Condition

Gilthead sea breams (*Sparus aurata*) were obtained from Aquaculture els Alfacs, S.L. (Les Cases d′Alcanar, Spain) and moved to the AQUAB fish facilities (Universitat Autònoma de Barcelona, UAB). Prior to the beginning of the experiment, the fish were acclimatized for three weeks at 20 °C. The fish included in the analysis were in the adult developmental stage, weighing 100 ± 5.0 g. During the experiment, the fish were subjected to a 12 L:12 D photoperiod in a closed recirculation system and fed with commercial diets (Skretting). The water parameters were monitored daily and maintained at basal levels, including dissolved oxygen, pH, ammonia, nitrites, nitrates, and salinity. The experiment complied with the Guiding Principles for Biomedical Research Involving Animals (EU2010/63/EU) and the guidelines of the Spanish laws (law 32/2007 and RD 53/2013) and was approved by the Committee on the Ethics of Animal Experiments of the University of Murcia (Permit Number: A13150104).

### 2.2. Experimental Design

The fish were subjected to a superficial skin lesion using a disposable circular biopsy punch stainless steel with a diameter of 4 mm (Quirurgimedical, Viña del Mar, Chile). The fish were sedated with 20 mg L^−1^ clove oil for the superficial wound incision procedure in the skin. The mechanical damage was applied with the fish facing to the left. All the lesions were located in the same skin region (behind the dorsal fin) and applied above (IU group) or below (ID group) the lateral line. The control group (fish with no lesions) underwent the same handling process as the experimental groups. Each experimental group was separated into a different tank (*n* = 15 fish per tank). The fish were sampled at 2-, 4- and 20-days post-injury (dpi). No mortalities were recorded during the experimental trial.

### 2.3. Sampling

Fish (*n* = 3 per treatment and time point evaluated) were randomly taken from each tank and sacrificed by over-anesthetization in MS222 (200 mg L^−1^). A skin tissue quadrant was collected around the damaged area of the tissue where the wound was made (Injury Up (IU); Injury Down (ID)), taking care to avoid the presence of muscle tissue in the sample. Afterward, the samples were frozen in liquid nitrogen and stored at −80 °C until analysis.

### 2.4. Isolation of RNA and cDNA Synthesis

A total of 3 fish per treatment and time point evaluated were subjected to total RNA isolation from individual fish skins using TRI Reagent (Sigma, St. Louis, MO, USA), according to the manufacturer’s instructions. The total RNA pellet was dissolved in nuclease-free water and quantified using a NanoDrop ND-2000 spectrophotometer (Thermo Scientific, Waltham, MA, USA). All samples were immediately stored at −80 °C until use. The total RNA (2 μg) was used as a template to synthesize complementary DNA (cDNA) using an iScript cDNA kit (Bio-Rad Laboratories, Hercules, CA, USA), according to the manufacturer’s instructions.

### 2.5. Quantitative Real-Time PCR (qPCR)

Skin samples from fish were analyzed using real-time PCR. The analysis included the evaluation of genes encoding neuropeptides, including *corticotropin-releasing hormone* (*crh*), *galanin* (*galn*), *growth hormone-releasing hormone* (*ghrh*), *neuropeptide B* (*npb*), *neuropeptide Y* (*npy*), *proenkephalin-B* (*penkb*), and *tachykinin 1* (*tac1*). The hormone-encoding genes included *angiotensinogen* (*agt*), *cholecystokinin* (*cck*), *glucagon-1* (*gcga*), *glucagon-2* (*gcgb*), *leptin* (*lep*), *oxytocin* (*oxt*), *pro-opiomelanocortin-A* (*pomca*), *prolactin* (*prl*), *somatostatin-1B* (*sst1b*), and *vasoactive intestinal peptide* (*vip*). Finally, the group of genes encoding modulators related to the tissue repair modulators included the *epidermal growth factor receptor* (*egfr*), *pro-epidermal growth factor* (*pro-egf*), *interleukin-6* (*il-6*), *vascular endothelial growth factor A* (*vegfa*), and *vascular endothelial growth factor C* (*vegfc*). The genes were selected considering their relevance in regulating the HPA and HPI axes in teleost skin. Several reference genes, *elongation factor 1-alpha 1* (*ef1α*), *ribosomal protein L27* (*rpl27*), and *ribosomal subunit 18* (*18s*), were tested to elucidate which one had less variation and, consequently, was the best reference gene candidate for this experiment. The specific primers used for gilthead sea bream are indicated in Table 1. Complete gene sequences were obtained from three genome databases; the first was a database discovered after a search by Ensembl: EGENOME T20103; the second was a transcriptomic database provided by the Department of Cell Biology and Histology of the Faculty of Biology (University of Murcia); and the third was GenBank, the nucleotide sequence database of the NIH (National Institute of Health). Subsequently, the similarity with known orthologues was analyzed using the BLAST program through the Bioinformatics Resources Portal of the SIB ExPASy. Thus, the amplicon sequence was compared against all known sea bream genomes and transcriptomes to verify their unique sequences. Subsequently, primers were designed using the applied online Biosystems™ Primer Designer™ Tool by ThermoFisher Scientific. The primer secondary structure and annealing specificity was checked with OligoAnalyzer (version 3.1) and Primer-Blast webtool (NCBI, https://www.ncbi.nlm.nih.gov/tools/primer-blast/), respectively. The undesirable PCR product appearance was previously verified by a single peak in the melting curve for each primer set. Real-time PCR reactions were performed with iTaq universal SYBR green supermix (Bio-Rad Laboratories) using a 1:10 cDNA dilution. The primers for all genes were used at a final concentration of 500 nM. The thermal conditions were 3 min at 95 °C of pre-incubation followed by 40 cycles at 95 °C for 30 s and 60 °C for 30 s. All the reactions were performed in duplicate using the CFX384 Touch Real-Time PCR Detection System (Bio-Rad Laboratories). The quantification was performed using the Livak method [23]. The values for each experimental condition were expressed as a normalized relative expression (NRE) using *18s* as a reference gene and the control group (fish with no skin lesion) as a calibrator in each time point evaluated. The results are expressed as the mean value obtained for the same treatment and time points evaluated. The data are expressed as the mean ± SD and are displayed in Table S1 (neuropeptides), Table S2 (hormones), and Table S3 (tissue repair modulators).

### 2.6. Statistical Analysis

The results were expressed as the mean ± SD (*n* = 3 fish per treatment and time point evaluated). All data were analyzed using a two-way ANOVA followed by Tukey’s post hoc analysis to determine differences among groups and times. All the statistical analyses were conducted using GraphPad Prism (version 9.0), and differences were considered statistically significant when *p* < 0.05 among groups.

## 3. Results

**Figure 1:**
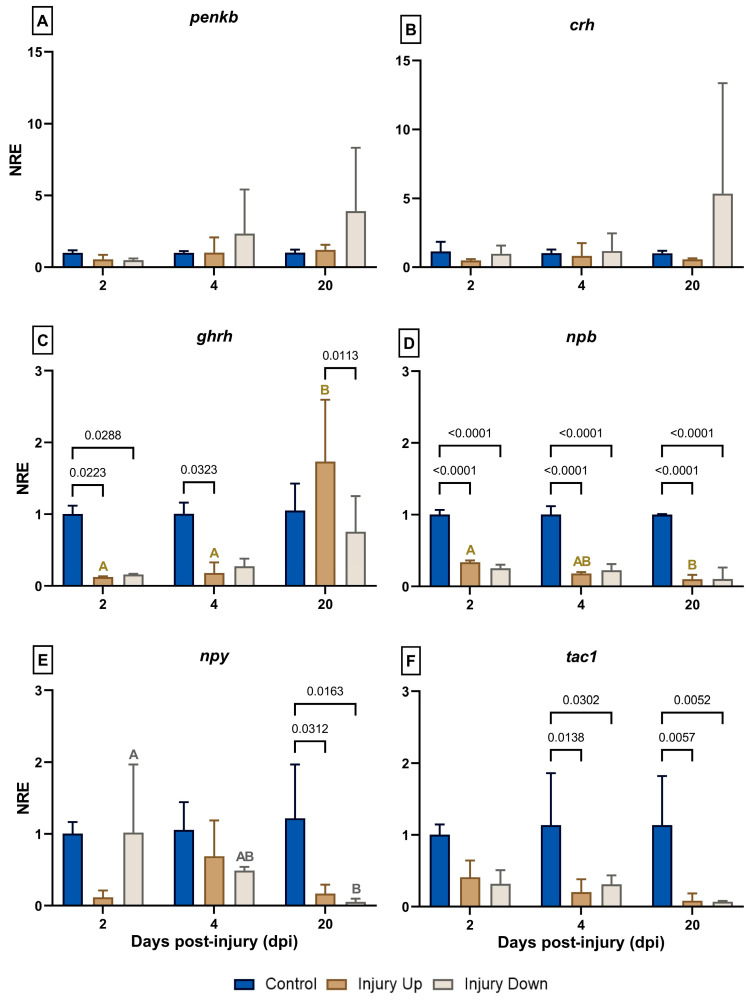
Normalized relative expressions (NREs) of genes associated with neuropeptides in the skin of gilthead sea bream following mechanical skin damage. Gene expression analysis was performed using real-time quantitative polymerase chain reaction (RT-qPCR). The results are expressed as the NRE and normalized using *18s* as a reference gene and the control group (fish with no skin lesion) as a calibrator at each sampling time. The NRE was calculated according to the Livak formula [23]. (**A**) *Proenkephalin-B* (*penkb*), (**B**) *corticotropin-releasing hormone* (*crh*), (**C**) *growth hormone-releasing hormone* (*ghrh*), (**D**) *neuropeptide B* (*npb*), (**E**) *neuropeptide Y* (*npy*), and (**F**) *tachykinin 1* (*tac1*). Sampling was performed at 2-, 4-, and 20-days post-injury (dpi). The experimental groups are defined as the IU group (injury up the lateral line) or ID group (injury down the lateral line). Data are expressed as the mean ± SD (*n* = 3 per treatment and evaluated time point). Two-way ANOVA and Tukey’s post hoc multiple comparison tests were used to determine statistical differences among groups (α = 0.05). The values of the significant differences between the groups at each time point are indicated, and the different capital letters denote significant differences over time for the different groups.

The expressions of several neuropeptide genes were evaluated (Figure 1). Among them, the expressions of *penkb* (Figure 1A) and *crh* (Figure 1B) registered no variations in any of the treatments at any of the time points assessed. However, at 20 dpi, a significant increase in the NRE mean value in the IU group was observed when compared to the injury time. In the case of the ID group, an increasing trend was registered compared to the control group, although with no significance probably due to its high variability (NRE_ID; 20dpi_ = 5.34 ± 8.01). By contrast, no amplification was obtained for *galn* in any of the samples analyzed.

The *ghrh* expression presented a downregulation of the IU group at 2 dpi (NRE_IU; 2dpi_ = 0.123 ± 0.012) and 4 dpi (NRE_IU; 4dpi_ = 0.18 ± 0.147) compared to the control group (NRE_CG; 2dpi_ = 1.003 ± 0.117 and NRE_CG; 4dpi_ = 1.007 ± 0.155, respectively) (Figure 1C). On the other hand, the ID group showed a similar expression pattern, although it was only significantly downregulated at 2 dpi (NRE_ID; 2dpi_ = 0.16 ± 0.01) compared to the control group. Importantly, the IU at 20 dpi (NRE_IU; 20dpi_ = 1.733 ± 0.863) showed a marked increase in a time- (NRE_IU; 4dpi_ = 0.18 ± 0.147) and treatment-dependent manner (NRE_CG; 20dpi_ = 1.05 ± 0.377; NRE_ID; 20dpi_ = 0.757 ± 0.496).

When the expression of *npb* was assessed, a significant decrease compared to the respective control group was observed at all the analyzed time points (Figure 1D). In particular, at 2 dpi, the IU (NRE_IU; 2dpi_ = 0.337 ± 0.025) and ID (NRE_ID; 2dpi_ = 0.253 ± 0.050) groups were downregulated compared to the control group (NRE_CG; 2dpi_ = 1.003 ± 0.064). The same modulation was observed at 4 dpi (NRE_IU; 4dpi_ = 0.180 ± 0.020; NRE_ID; 4dpi_ = 0.227 ± 0.085) and at 20 dpi (NRE_IU; 20dpi_ = 0.1 ± 0.062; NRE_ID; 20dpi_ = 0.103 ± 0.162) compared to the control (NRE_CG; 4dpi_ = 1.003 ± 0.116; NRE_CG; 20dpi_ = 1.0 ± 0.01). In this downregulating scenario, the expression at 20 dpi in the IU group (NRE_IU; 20dpi_ = 0.1 ± 0.062) was even significantly lower than at 2 dpi (NRE_IU; 2dpi_ = 0.337 ± 0.025).

Regarding *npy*, a high variability was observed in general between the experimental groups for each time evaluated (Figure 1E). As a result, no significant differences were observed between the experimental groups at 2 dpi and 4 dpi. By contrast, at 20 dpi, the control group (NRE_CG; 20dpi_ = 1.22 ± 0.74) showed significant differences in comparison with the decreased values for IU (NRE_IU; 20dpi_ = 0.17 ± 0.12) and ID (NRE_ID; 20dpi_ = 0.05 ± 0.04). In the ID group, differences were only recorded between 2 dpi (NRE_ID; 2dpi_ = 1.02 ± 0.94) and 20 dpi (NRE_ID; 20dpi_ = 0.05 ± 0.04).

The expression of *tac1* showed a tendency toward downregulation in the IU and ID treatments compared to the control (Figure 1F) at 2 dpi. In the UI group, this downregulation was significant at 4 dpi (NRE_IU; 4dpi_ = 0.203 ± 0.180) and at 20 dpi (NRE_IU; 20dpi_ = 0.083 ± 0.101). The same modulation was observed in the ID group at 4 dpi (NRE_ID; 4dpi_ = 0.203 ± 0.180) and at 20 dpi (NRE_ID; 20dpi_ = 0.070 ± 0.010).

**Figure 2:**
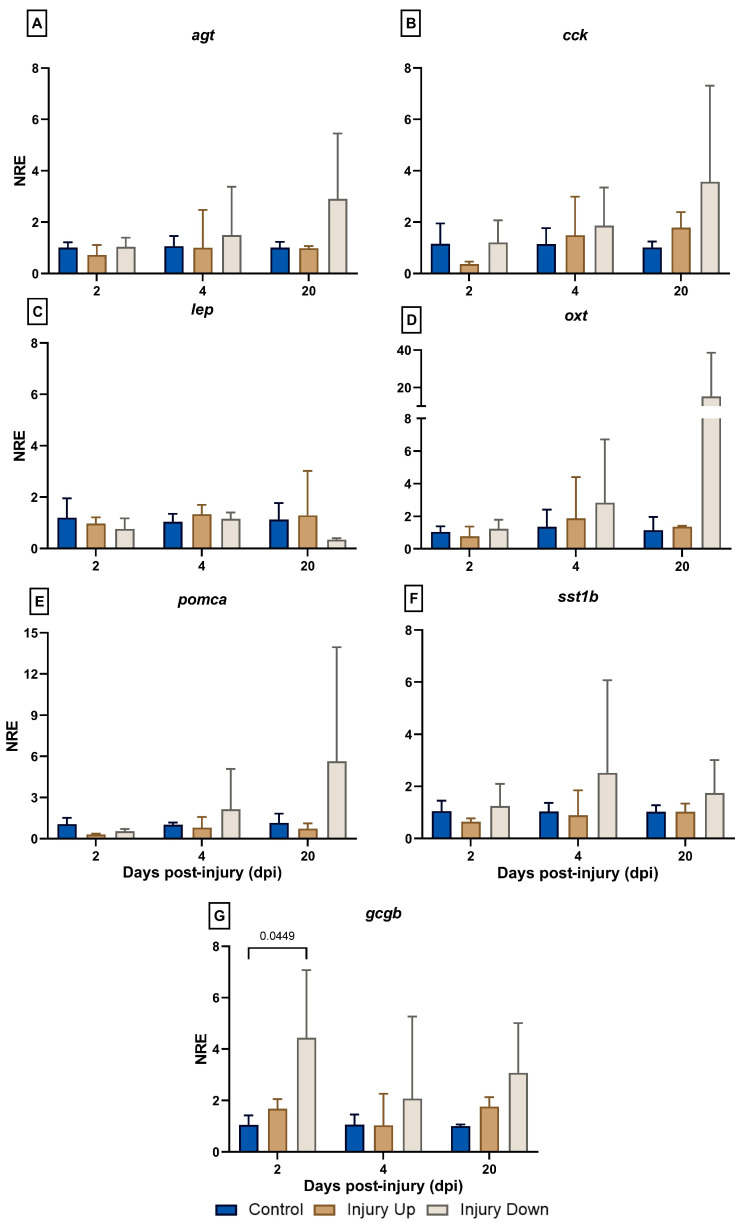
Normalized relative expressions (NREs) of genes associated with hormones in the skin of gilthead sea bream following mechanical skin damage. Gene expression analysis was performed using real-time quantitative polymerase chain reaction (RT-qPCR). The results are expressed as the NRE and normalized using *18s* as a reference gene and the control group (fish with no skin lesion) as a calibrator at each sampling time. The NRE was calculated according to the Livak formula [23]. (**A**) *Angiotensinogen* (*agt*), (**B**) *cholecystokinin* (*cck*), (**C**) *leptin* (*lep*), (**D**) *oxytocin* (*oxt*), (**E**) *pro-opiomelanocortin*-*A* (*pomca*), (**F**) *somatostatin-1B* (*sst1b*), and (**G**) *glucagon-2* (*gcgb*). Sampling was performed at 2-, 4-, and 20-days post-injury (dpi). The experimental groups are defined as the IU group (injury up the lateral line) or ID group (injury down the lateral line). Data are expressed as the mean ± SD (*n* = 3 per treatment and evaluated time point). Two-way ANOVA and Tukey’s post hoc multiple comparison tests were used to determine statistical differences among groups (α = 0.05). The values of the significant differences between the groups at each time point are indicated.

The hormone expression profiling registered no modulation or amplification in most of the genes assessed (Figure 2). Thus, the expressions of *agt*, *cck*, *lep*, *oxt*, *pomca*, and *sst1b* registered no variations in any of the treatments and time points (Figure 2A–F), while no amplifications were obtained for *gcga*, *prl*, and *vip* in any of the samples analyzed. The expression of *gcgb* registered an increasing trend in the IU group at 2 dpi (NRE_ID; 2dpi_ = 4.44 ± 2.629) compared to the control group (NRE_CG; 2dpi_ = 1.043 ± 0.379). However, none of the analyzed groups showed differences in the expression kinetics (Figure 2G).

**Figure 3:**
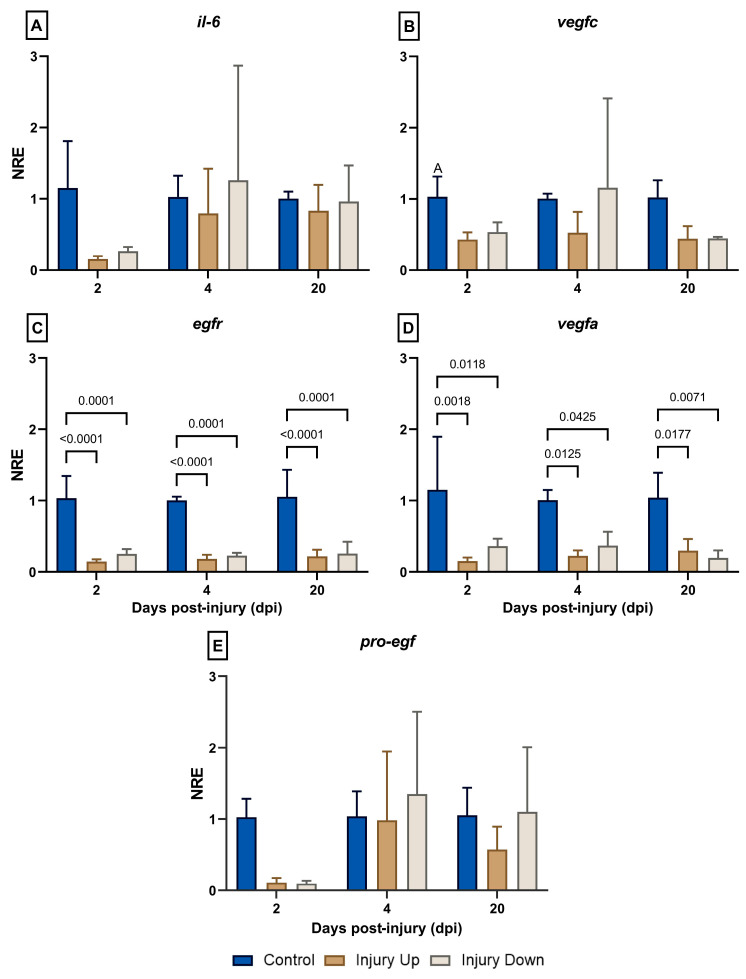
Normalized relative expression (NRE) genes associated with tissue repair in the skin of gilthead sea bream following mechanical skin damage. Gene expression analysis was performed using real-time quantitative polymerase chain reaction (RT-qPCR). The results are expressed as the NRE and normalized using *18s* as a reference gene and the control group (fish with no skin lesion) as a calibrator at each sampling time. The NRE was calculated according to the Livak formula [23]. (**A**) *Interleukin-6* (*il-6*), (**B**) *vascular endothelial growth factor C* (*vegfc*), (**C**) *epidermal growth factor receptor* (*egfr*), (**D**) *vascular endothelial growth factor A* (*vegfa*), and (**E**) *pro-epidermal growth factor* (*pro-egf*). Sampling was performed at 2-, 4-, and 20-days post-injury (dpi). The experimental groups are defined as the IU group (injury up the lateral line) or ID group (injury down the lateral line). Data are expressed as the mean ± SD (*n* = 3 per treatment and evaluated time point). Two-way ANOVA and Tukey’s post hoc multiple comparison tests were used to determine statistical differences among groups (α = 0.05). The values of the significant differences between the groups at each time point are indicated.

Regarding the modulation of immune-related genes (Figure 3), the expressions of *il-6* and *vegfc* registered no variations in any of the treatments (Figure 3A,B). The expression of *egfr* showed a significant decrease in the IU and ID experimental groups at 2 dpi (NRE_IU; 2dpi_ = 0.14 ± 0.03; NRE_ID; 2dpi_ = 0.25 ± 0.07), 4 dpi (NRE_IU; 4dpi_ = 0.18 ± 0.06; NRE_ID; 4dpi_ = 0.22 ± 0.04), and 20 dpi (NRE_IU; 20dpi_ = 0.21 ± 0.09; NRE_ID; 20dpi_ = 0.25 ± 0.16) compared to the control group at each assessed time (NRE_CG; 2dpi_ = 1.03 ± 0.31; NRE_CG; 4dpi_ = 1.00 ± 0.05; NRE_CG; 20dpi_ = 1.05 ± 0.38) (Figure 3C). The expression of *vegfa* showed a pattern of expression very similar to that recorded for *egfr* (Figure 3D). Finally, in the case of *pro-egf*, a tendency to decrease in its expression (although not significant) was observed at 2 dpi in the IU (NRE_IU; 2dpi_ = 0.11 ± 0.06) and ID groups (NRE_ID; 2dpi_ = 0.09 ± 0.04) compared to the control group (NRE_IU; 2dpi_ = 1.02 ± 0.26) (Figure 3E). By contrast, an increase in the expression kinetics in the ID group was observed at 4 dpi (NRE_ID; 4dpi_ = 1.35 ± 1.15) compared to 2 dpi (NRE_ID; 2dpi_ = 0.09 ± 0.04). However, no significant differences were seen at 4 dpi between the IU (NRE_IU; 4dpi_ = 0.98 ± 0.96) and CG groups (NRE_CG; 4dpi_ = 1.03 ± 0.35). No differences were recorded at 20 dpi between the different experimental groups (NRE_CG; 20dpi_ = 1.05 ± 0.38; NRE_IU; 20dpi_ = 0.57 ± 0.32; NRE_ID; 20dpi_ = 1.10 ± 0.90).

## 4. Discussion

The skin of bony fish is one of the most physically resistant organs to external aggressions and is responsible for maintaining the integrity of the fish. Skin lesions act as entry sites for pathogens normally present in the aquatic environment. In this way, wound repair has barely been studied in sea bream [1,17], one of the most relevant species in the Mediterranean Sea in terms of volume produced and economic value [24]. Therefore, the analysis of the peripheral neuro-endocrine and tissue repair responses is of great interest to determine the communication of these regulatory systems when the skin of the sea bream is subjected to mechanical damage by incision, resulting in a superficial wound. Our results in sea bream show that the gene expression profile depends on the site of the superficial wound (above or below the lateral line). Importantly, previous studies also registered differences depending on the skin region analyzed. In fact, Cordero and collaborators [17,22] reported that the ventral region undergoes wound healing better and faster than a wound in the dorsal area. Furthermore, the comparison between both skin regions also showed differences in the gene expression patterns of a set of cytokines that play a key role in the activation and regulation of the immune response (*il1b*, *tnfα*, *il6*, *il7*, *il8*, *il18*, *il10*, and *tgfβ*), demonstrating greater modulation and susceptibility of the ventral than the dorsal area. In the same line, another study also showed a higher expression of *ight* (the IgT heavy chain gene, a mucosal-specialized immunoglobulin [9]) on the ventral than the dorsal region of sea bream skin [20]. Differences were also visualized using macroscopic scanning electron microscopy, confirming faster healing of the ventral wound [1,17]. Likewise, the enzymatic activities of peroxidase and esterase, two important microbicidal agents, presented higher values in the skin mucus of the fish 2 and 3 days after the ventral wound. 

Differences in the expression profiles between areas of the skin may be associated with anatomical and histological characteristics. Histologically, the ventral region has a greater epidermis thickness than the dorsal skin [17]. In addition, the apical part of the dorsal epidermal cells has a larger cell size and microgroove area than the skin’s ventral epidermal cells [21]. In fact, these characteristics suggest that the dorsal area is associated with protection mechanics and has a greater capacity to retain skin mucus, thus improving the mucosal immune-barrier function [17]. Taken together, these anatomical, transcriptional, and enzymatic differences suggests that the skin cells of sea bream are more sensitive to physical attacks in the area below the lateral line.

In vertebrates, it is assumed that there is a complex communication between the neuroimmune and endocrine systems in the skin, comprising a network of common chemical messengers and their receptors, which interact through a combination of endocrine, paracrine, and/or autocrine mechanisms to exert pleiotropic effects that favor homeostasis [25]. Among these chemical messengers and receptors, we have analyzed the roles of some of them in the growth, tissue repair, stress, and signaling processes.

The growth hormone (GH), a member of the somatotropic axis, is among the messengers usually shared between the endocrine, nervous, and immune systems. Although pituitary GH expression is regulated by several hypothalamic neuropeptides, such as growth hormone-releasing hormone (GHRH) [26], it remains unclear how extrapituitary GH expression is regulated [27]. The endocrine functions of GH include angiogenesis, metabolic regulation, and homeostasis [28]. While the functional relevance of GH in the immune system is through the presence of the GH receptor in immune cells, including lymphocytes, macrophages, or dendritic cells [29,30], the neuroregulation of GH secretion is mediated in part by GHRH, one of the most important factors to stimulate the growth hormone in mammals [31,32], which is mainly produced in the pituitary gland [33]. Although it has been reported that in sea bream, the lesion below the lateral line heals after 15 days [17], there is no accurate record of the healing of an upper lesion in this species. In our study, GHRH presented a downregulation on day 2 post-injury compared to the control, which was still observed on day 4 post-injury in both damaged areas (lesion up and down of the lateral line). This modulation in the first 4 days after mechanical damage could be due to an effect derived from the stress condition produced by the skin wound. Thus, in mammals, stress has been reported to reduce immune-induced GHRH levels [34]. However, on day 20, the expression of *ghrh* at the lesion seems to depend on the location. In fact, in our study, the lesion above the lateral line increased the *ghrh* expression compared with the lesion below the lateral line, which showed closer levels to the control values. This result could be associated with the activation of the tissue repair-related process on the lesion below the lateral line. In this scenario, the increase in the *ghrh* expression at 20 days in the lesion above the lateral line could be due to the stressful condition that kept it initially downregulated and overcoming after 4 days. 

CRH, apart from its GH-releasing action, has a releasing action on pro-opiomelanocortin peptides (POMC), which, in turn, activate the production of adrenocorticotropin (ACTH) [35,36]. It has been shown in humans that locally produced CRH is involved in the inflammatory process, stimulating angiogenesis in vivo and endothelial cell chemotaxis in vitro. In line with this idea, CRH mRNAs and peptides have been identified in the skin [37]. In mammals, immune system cells can synthesize and secrete neuroendocrine hormones such as GH, GHRH, CRH, and ACTH [34]. As immune cells produce significantly less of these hormones than the pituitary, hypothalamus, and liver, their local production acts through paracrine/autocrine or intracrine mechanisms, supporting the immune response. According to this idea, in our study, we observed a trend of progressive increase in the expressions of the *crh*, *pomca*, and *cck* genes in sea bream with lesions below the lateral line. These genes may indirectly participate in releasing GH, inflammatory processes, or stimulating angiogenesis. Neurons releasing CRH are located in the neurosecretory preoptic area (NPO), forming an interspersed group with other neurons that express *oxytocin* (*oxt*), *arginine vasopressin* (*avp*), *proenkephalin a* (*penka*), *neurotensin* (*nts*), and *somatostatin* (*sst1.1*). Another separate NPO subregion of neurons expresses *cholecystokinin* (*cck*), *proenkephalin b* (*penkb*), and *vasoactive intestinal peptide* (*vip*). Within the first NPO group, *crh* shows low co-expression with *penk-a* and *penk-b* [38,39,40]. Importantly, the same phenomenon is observed in our study, with the increasing trend of *crh* expression and its co-expression with *penkb*, *cck*, *oxt*, *pomca*, and (less steep) *sst1b*. OXT is a neuropeptide hormone that participates in wound healing and is required for skin repair after injury, including re-epithelialization, collagen, and fibrinogenesis [41]. In addition, the relationship between these is more evident in the fish that presented the lesion below the lateral line. *Angiotensinogen* (*agt*) is another gene that, in our study, showed a similar trend to *crh* and its co-expression with *penkb*, *cck*, *oxt*, *pomca*, and *sst1b* in fish with the lesion below the lateral line. AGT is the sole precursor of all angiotensin peptides, which are essential for the renin–angiotensin hormonal function, which is a crucial regulator of blood pressure homeostasis in keratinocyte re-epithelialization in peripheral tissues and angiogenesis during wound healing in mammals [42,43]. These results suggest that a neuroendocrine network is activated in response to skin tissue repair caused by mechanical damage.

In response to the skin wound, a pain reaction would be expected as a defense mechanism in response to the stimulus, perceived as a threat to the organism. In line with this idea, in our study, we evaluated the expressions of *neuropeptide B* (*npb*), *neuropeptide Y* (*npy*), and *tachykinin 1* (*tac1*). In mammals, there is evidence that NPB is involved in the modulation of inflammation, pain sensation, and behavior [44,45]. Importantly, no studies have reported the function of NPB in teleosts [46]. Similarly, TAC1 in mammals is involved in various biological actions commanded by the CNS, such as pain transmission, emotional behavior, learning, and memory [47]. Like *npy*, it is expressed in several tissues, including the skin, and it is involved in several functions, including the regulation of appetite, vasoconstriction, and pain. It can facilitate wound repair, a process dependent on angiogenesis [48,49,50,51]. In our study, the downregulation tendencies of *npb* and *tac1*, both in the lesions above and below the lateral line from 2 dpi and even more from 4 to 20 dpi, make us hypothesize that the nociception transmission signal could be activated at the earlier time points. In the case of *npy*, our results also support the hypothesis of a higher expression related to genes associated with pain and their transmission at earlier times. These pain indicators, such as *tac1* and *npy,* would act locally and earlier, as observed mainly in the lesion below the lateral line, and could lead to a local proangiogenic response by *vegf*. However, only at day 4 post-injury, the fish with the lesion above the lateral line showed a slightly higher *vegfc* expression, although not significant, than the control group. It is suggested that the expression of *vegf* could also have a local proangiogenic effect, since VEGF is a highly conserved protein family among animals evolutionarily so distant as fish and mammals [52,53]. Together with other global proangiogenic modulators such as GH and CRH, these molecules would be crucial to guide and coordinate the growth of blood vessels [48]. Since vascular repair is dependent on angiogenesis, new blood vessels can emerge as early as 3 days after injury, so the present study in sea bream suggests that the moderate increase observed for *vegfc*, together with the increase in the global proangiogenic modulator *crh* on day 20, could provide a conduit for nutrients and other healing mediators and a mechanism for the elimination of accumulated metabolites, shortening healing times for the lesion below compared to the lesion above.

Physiological responses to acute stress result from activating a series of pathways to a variety of adverse stimuli, including injury and infection [7]. Glucagon is one of the hormones also involved in the energetic and metabolic aspects associated with the activation of the stress response. The increase in glucagon leads to increased glucose availability, an adaptive response to situations requiring intense energetic effort, such as fighting, fleeing, or repairing injuries [54]. In our study, the increase in *glucagon* (*gcgb*) expression was observed at day 2 post-injury, suggesting that glucose requirements are still high two days after the injury. A study on sea bream observed that *cgc* also acts on adipose tissue as a powerful lipolytic hormone [55]. In line with this antecedent, we registered the upregulation of *gcgb* (although not significant) at 4 and 20 dpi, suggesting that it would be related to the restoration of adipose tissue in the repair of the injury.

The pro-inflammatory genes play a crucial role in response to damage to the epithelial barrier and in preventing a potential infection of microorganisms present in the damaged tissue. In our study, we evaluated the expressions of *il-6* and the *pro-epidermal growth factor* (*pro-egf*), both pro-inflammatory inducers that showed a pretty similar expression trend. In no one fish did we observe a differential expression in any of the treatments evaluated. These results contrast with the lower expression of the *egf receptor* (*egfr*) at all the times assessed. Methodologically, as a time of less than 2 days post-injury was not included in our experimental design, the modulation of these genes at earlier time points should be evaluated in future studies. In mammals, it has been observed that after the injury, there is an early release of IL-6 that induces the release of pro-inflammatory cytokines from M1 macrophages (IL-6, IL-1β, IL-4, and TNF-α), keratinocytes, endothelial cells, stromal cells that reside in tissues and induce Th17 (pro-inflammatory), and Th2 (anti-inflammatory, chemotaxis, and differentiation in CD4^+^) cells. IL-6, together with VEGF, leads to neovascularization, which supports fibroproliferative scarring [56]. On the other hand, the pro-EGF is a type 1 membrane protein that, after insertion into the membrane and following stimulation, becomes a functional EGF peptide hormone, which activates the EGF receptor (EGFR), playing an important role in regulating wound healing, growth, survival, migration, apoptosis, proliferation, and cellular differentiation and is a potent mitogenic factor for a variety of ectodermal and mesodermal cell types and tissues, including skin keratinocytes [57,58,59,60]. Another critical aspect of the presence of EGF in mammals is that it has been observed in external biological fluids such as saliva or tears, suggesting that EGF secretion is mainly an exocrine secretory process [61]. Meanwhile, as the inflammation progresses, *il-6* expression decreases significantly during the remodeling phase, possibly due to the apoptosis of infiltrating inflammatory leukocytes [56]. Further studies must include more time points to unveil the fine progression of these pro-inflammatory genes in response to skin injury in fish.

Further studies are necessary to understand the modulation of the genes involved in peripheral neuro-endocrine and tissue repair responses to mechanical damage in the skin of teleost fish. The evaluation of other genes analyzed in previous studies, such as those associated with tissue repair and other processes like the immune response [21,22], will provide an interesting point of view about how different but complementary biological processes coexist to promote the recovery of the skin wound. In terms of the dynamic of the response, the evaluation of more time points in the experimental design will provide a clearer perspective on expression dynamics at dorsal and ventral lesions, particularly of those markers that, in our study, only showed downregulation or an increased trend (but not significant). In this way, the analysis of the activation of the stress response and transcriptional activity at earlier times (i.e., 1-, 6-, and 24-hours post-injury as a measure to evaluate the immediate effect of the stimulus [4,62,63]) will shed light on the mode of action orchestrated by different systems to ensure tissue repair, defense, and protection of the organism.

## 5. Conclusions

To conclude, our study in sea bream is the first to analyze a considerable number of genes related to the peripheral neuro-endocrine and tissue repair responses of skin subjected to a superficial wound lesion, in addition to comparing the reaction between a wound above or below the lateral line. We showed statistically significant differences when comparing the location of the lesion, presenting a higher magnitude of expression in the group of fish with the lesion below compared to the group with the lesion above the lateral line. Enriching the knowledge of previous works regarding the thickness of a lesioned skin epidermis, we identify molecular agents that are biologically relevant in the repair of the injury. Overall, the results also suggest that a wound below the lateral line may be more critical due to its proximity to vital organs and susceptibility to infection by pathogens. The results also suggest a prompt and more robust repair response for injuries below than those above the lateral line.

## Figures and Tables

**Table 1:** List of primers for RT-qPCR analysis.

Biological Function	Gene Name	Gene Acronym	GenBank Accession Number	Primer Sequence (5′→3′)	Amplicon Size (bp)
Neuropeptides	*Corticotropin-releasing hormone*	*crh*	KC195964	FW: TCTTCGTCCATGTATCCCGG RV: AGCAGGTGGAAGGTCAGATC	203
	*Galanin*	*galn*	T20103:4520	FW: AGGAGGACTTCAGAACAGGC RV: TGTCCAAGGCTCCAATCTCT	108
	*Growth hormone-releasing hormone*	*ghrh*	DQ659328	FW: TGATGGCAAAACGTGTAGGC RV: CCGGCGTCCTTTGTTTCTAA	188
	*Neuropeptide B*	*npb*	Spau1B013495	FW: CATCCTCAAAAGCATGGCCA RV: CCAGGGTGAGGAAGACGTC	119
	*Neuropeptide Y*	*npy*	Spau1B023653	FW: GCCAAGTACTACTCAGCCCT RV: ATCTCGACTGTGGAAGGGTG	145
	*Proenkephalin-B*	*penkb*	Spau1B016802	FW: GAGAGAGGTGTAGACGAGCC RV: GCTTGTCCCACTTCAGGTTG	226
	*Tachykinin 1*	*tac1*	T20103:14549	FW: TCATTGGGAAGGACTCAGCA RV: ACATGGCTCCTTGATCCTCG	108
Hormones	*Angiotensinogen*	*agt*	T20103:4928	FW: CCTACGGATCCCTCTTCACC RV: CGTCGTCCACCAGAGAGTTA	171
	*Cholecystokinin*	*cck*	Spau1B015900	FW: TCCCTACCAGACCAGATCCT RV: TCCAAAGTCCATCCAGCCAA	100
	*Glucagon-1*	*gcga*	Spau1B008631	FW: ATGTAGACGGGAGCTTCACC RV: ATTGGCCCGCTTGTCTTTTC	126
	*Glucagon-2*	*gcgb*	Spau1B020957	FW: ATGTAGACGGGAGCTTCACC RV: ATTGGCCCGCTTGTCTTTTC	100
	*Leptin*	*lep*	Spau1B029280	FW: CTCGGGCTGATGATCTGGAT RV: CGTGCTTGATCTGTGAGACG	105
	*Oxytocin*	*oxt*	Spau1B008906	FW: GAGAACTACCTGCTCACCCC RV: CAGTCAGAGTCCACCGTACA	119
	*Pro-opiomelanocortin-A*	*pomca*	HM584909	FW: GATGATGAGAAGGCGGAGGA RV: TGGCTCCTGTCCATCTTTGT	216
	*Prolactin*	*prl*	Spau1B018970	FW: AGAGAATGGCGAGACAGGAG RV: AGTTGTTGAAGTCATGGTGGTG	114
	*Somatostatin-1B*	*sst1b*	Spau1B024306	FW: GTGTCTGGCTTGTTGGATGG RV: TAGACAGCCCTCTCCTCCAG	110
	*Vasoactive intestinal peptide*	*vip*	Spau1B010730	FW: CAGACAACTACAGCCGCTTC RV: GTTCGTCCCTGGATTCCTCA	123
Tissue repair modulators	*Epidermal growth factor receptor*	*egfr*	Spau1B012402T1	FW: GGAGGCCGTCATGAACAAAAC RV: CGTATTTCCACACCAGCGCA	191
	*Pro-epidermal growth factor*	*pro-egf*	Spau1B019439T1	FW: TCGATTTCACAGAGGACCGC RV: GGTCGCCCTACTTGGTTCTC	115
	*Interleukin 6*	*il-6*	EU244588.1	FW: TCGCCCACTGTTGCATAAGT RV: ATGAATCAGCGGTCGGATCC	139
	*Vascular endothelial growth factor A*	*vegfa*	Spau1B002667T5	FW: CCAAGAGTATGTGTCAGCCCA RV: CCGCATTACCTGCAATGTGAC	182
	*Vascular endothelial growth factor C*	*vegfc*	Spau1B001969T1	FW: TCAGCAGATGTGTGTGGACC RV: GAGGTGTCGGTTAGAGCCAC	105
Reference genes	*Elongation factor 1 alpha*	*ef1a*	AF184170.1	FW: TGTCATCAAGGCTGTTGAGC RV: GCACACTTCTTGTTGCTGGA	115
	*Ribosomal protein S18*	*18s*	AM490061.1	FW: CGAAAGCATTTGCCAAGAAT RV: AGTTGGCACCGTTTATGGTC	102
	*Ribosomal protein L27*	*rpl27*	AY188520.1	FW: AAGAGGAACACAACTCACTGCCCCAC RV: GCTTGCCTTTGCCCAGAACTTTGTAG	160

## Data Availability

The original contributions presented in the study are included in the article/Supplementary Materials. Further inquiries can be directed to the corresponding authors.

## Supplementary Materials

The following supporting information can be downloaded at: https://www.mdpi.com/article/10.3390/ani14121815/s1, Table S1: Normalized relative expression (NRE) of genes associated with neuropeptides in the skin of gilthead sea bream following skin mechanical damage; Table S2: Normalized relative expression (NRE) of genes associated with hormones in the skin of gilthead sea bream following skin mechanical damage; Table S3: Normalized relative expression (NRE) of genes associated with the tissue repair modulators in the skin of gilthead sea bream following skin mechanical damage.
