# Supplementary material for: Transcriptional Evaluation of Neuropeptides, Hormones, and Tissue Repair Modulators in the Skin of Gilthead Sea Bream (Sparus aurata L.) Subjected to Mechanical Damage
Source: Animals (Basel). 2024 Jun 18;14(12):1815. doi: 10.3390/ani14121815 (PMC11200434; doi:10.3390/ani14121815)
Supplement: Supplementary file 1 [file animals-14-01815-s001.zip › animals-3015555-supplementary.pdf]

animals

Supplementary Materials:

Table S1: Normalized relative expression (NRE) of genes associated with neuropeptides in the skin of gilthead sea bream following skin mechanical damage. Gene expression analysis was performed using real-time quantitative polymerase chain reaction (RT-qPCR). The results are expressed as NRE and normalized using the *18s* as a reference gene and the control group (fish with no skin lesion) as a calibrator at each sampling time. NRE was calculated according to the Livak formula [23]. Sampling was performed at 2-, 4-, and 20-days post-injury (dpi). The experimental groups are defined as Control, Injury Up or Injury Down the lateral line. Data are expressed as mean ± SD (n = 3 per treatment and evaluated time point).

Biological function	Gene name	Gene acronym	Control			Injury Up			Injury Down		
			2 dpi	4 dpi	20 dpi	2 dpi	4 dpi	20 dpi	2 dpi	4 dpi	20 dpi
Neuropeptides	*Corticotropin Releasing Hormone*	*crh*	1.137±0.703	1.023±0.266	1.010±0.175	0.497±0.085	0.817±0.931	0.577±0.067	0.973±0.596	1.173±1.281	5.340±8.011
	Growth Hormone Releasing Hormone	*ghrh*	1.003±0.117	1.007±0.155	1.050±0.377	0.123±0.012	0.180±0.147	1.733±0.863	0.160±0.010	0.273±0.107	0.757±0.496
	Neuropeptide B	*npb*	1.003±0.064	1.003±0.116	1.000±0.010	0.337±0.025	0.180±0.020	1.000±0.062	0.253±0.050	0.227±0.085	0.103±0.162
	Neuropeptide Y	*npy*	1.007±0.161	1.057±0.389	1.220±0.748	0.117±0.096	0.690±0.500	0.170±0.125	1.020±0.949	0.490±0.052	0.053±0.045
	Proenkephalin-B	*penkb*	1.010±0.171	1.003±0.110	1.013±0.206	0.550±0.306	1.020±1.057	1.213±0.346	0.493±0.112	2.340±3.067	3.913±4.402
	Tachykinin 1	*tac1*	1.003±0.144	1.137±0.726	1.137±0.685	0.410±0.235	0.203±0.180	0.083±0.101	0.320±0.190	0.313±0.123	0.070±0.010

Table S2: Normalized relative expression (NRE) of genes associated with hormones in the skin of gilthead sea bream following skin mechanical damage. Gene expression analysis was performed using real-time quantitative polymerase chain reaction (RT-qPCR). The results are expressed as NRE and normalized using the *18s* as a reference gene and the control group (fish with no skin lesion) as a calibrator at each sampling time. NRE was calculated according to the Livak formula [23]. Sampling was performed at 2-, 4-, and 20-days post-injury (dpi). The experimental groups are defined as Control, Injury Up or Injury Down the lateral line. Data are expressed as mean ± SD (n = 3 per treatment and evaluated time point).

Biological function	Gene name	Gene acronym	Control			Injury Up			Injury Down		
			2 dpi	4 dpi	20 dpi	2 dpi	4 dpi	20 dpi	2 dpi	4 dpi	20 dpi
Hormones	*Angiotensinogen*	*agt*	1.013±0.206	1.063±0.403	1.017±0.220	0.727±0.384	1.007±1.468	0.987±0.083	1.040±0.357	1.500±1.879	2.907±2.548
	Cholecystokinin	*cck*	1.157±0.793	1.147±0.619	1.013±0.231	0.373±0.092	1.493±1.496	1.790±0.599	1.0210±0.862	1.863±1.485	3.570±3.744
	Glucagon-2	*gcgb*	1.043±0.379	1.057±0.397	1.000±0.066	1.683±0.369	1.033±1.227	1.760±0.369	4.440±2.629	2.067±3.195	3.073±1.934
	Leptin	*lep*	1.203±0.750	1.037±0.311	1.130±0.640	0.973±0.241	1.337±0.366	1.297±1.726	0.760±0.416	1.153±0.249	0.343±0.059
	Oxytocin	*oxt*	1.037±0.350	1.360±1.050	1.153±0.803	0.783±0.587	1.873±2.529	1.367±0.055	1.243±0.546	2.827±3.890	15.123±23.434
	Pro-opiomelanocortin-A	*pomca*	1.060±0.459	1.010±0.161	1.157±0.665	0.297±0.067	0.803±0.781	0.730±0.372	0.540±0.174	2.157±2.926	5.637±8.318
	Somatostatin-1B	*sst1b*	1.050±0.404	1.037±0.332	1.027±0.248	0.647±0.127	0.893±0.960	1.030±0.314	1.247±0.856	2.523±3.546	1.747±1.270

Table S3: Normalized relative expression (NRE) of genes associated with the tissue repair modulators in the skin of gilthead sea bream following skin mechanical damage. Gene expression analysis was performed using real-time quantitative polymerase chain reaction (RT-qPCR). The results are expressed as NRE and normalized using the *18s* as a reference gene and the control group (fish with no skin lesion) as a calibrator at each sampling time. NRE was calculated according to the Livak formula [23]. Sampling was performed at 2-, 4-, and 20-days post-injury (dpi). The experimental groups are defined as Control, Injury Up or Injury Down the lateral line. Data are expressed as mean ± SD (n = 3 per treatment and evaluated time point).

Biological function	Gene name	Gene acronym	Control			Injury Up			Injury Down		
			**2 dpi**	**4 dpi**	**20 dpi**	**2 dpi**	**4 dpi**	**20 dpi**	**2 dpi**	**4 dpi**	**20 dpi**
Tissue repair modulators	*Epidermal Growth Factor Receptor*	*egfr*	1.033±0.311	1.003±0.051	1.053±0.376	0.143±0.032	0.180±0.061	0.217±0.095	0.250±0.070	0.227±0.040	0.257±0.166
	Pro-Epidermal Growth Factor	*pro-egf*	1.027±0.261	1.037±0.355	1.053±0.388	0.110±0.066	0.983±0.966	0.573±0.320	0.097±0.042	1.353±1.155	1.103±0.906
	Interleukin 6	*il-6*	1.150±0.661	1.027±0.298	1.003±0.097	0.157±0.040	0.797±0.626	0.833±0.336	0.267±0.059	1.263±1.606	0.963±0.506
	Vascular Endothelial Growth Factor A	*vegfa*	1.150±0.747	1.007±0.142	1.040±0.350	0.150±0.050	0.223±0.078	0.297±0.163	0.360±0.105	0.367±0.198	0.193±0.107
	Vascular Endothelial Growth Factor C	*vegfc*	1.030±0.286	1.003±0.068	1.020±0.244	0.430±0.101	0.527±0.291	0.443±0.175	0.537±0.137	1.157±1.255	0.447±0.021
